# Differences in Motor Evoked Potentials Induced in Rats by Transcranial Magnetic Stimulation under Two Separate Anesthetics: Implications for Plasticity Studies

**DOI:** 10.3389/fncir.2016.00080

**Published:** 2016-10-06

**Authors:** Matthew Sykes, Natalie A. Matheson, Philip W. Brownjohn, Alexander D. Tang, Jennifer Rodger, Jonathan B. H. Shemmell, John N. J. Reynolds

**Affiliations:** ^1^Brain Health Research Centre and Brain Research New Zealand Centre of Research ExcellenceDunedin, New Zealand; ^2^Department of Anatomy, University of OtagoDunedin, New Zealand; ^3^Experimental and Regenerative Neuroscience, School of Animal Biology, University of Western AustraliaPerth, WA, Australia; ^4^School of Physical Education, Sport and Exercise Sciences, University of OtagoDunedin, New Zealand

**Keywords:** transcranial magnetic stimulation, anesthesia, rat, motor evoked potential, plasticity, excitability

## Abstract

Repetitive transcranial magnetic stimulation (rTMS) is primarily used in humans to change the state of corticospinal excitability. To assess the efficacy of different rTMS stimulation protocols, motor evoked potentials (MEPs) are used as a readout due to their non-invasive nature. Stimulation of the motor cortex produces a response in a targeted muscle, and the amplitude of this twitch provides an indirect measure of the current state of the cortex. When applied to the motor cortex, rTMS can alter MEP amplitude, however, results are variable between participants and across studies. In addition, the mechanisms underlying any change and its locus are poorly understood. In order to better understand these effects, MEPs have been investigated *in vivo* in animal models, primarily in rats. One major difference in protocols between rats and humans is the use of general anesthesia in animal experiments. Anesthetics are known to affect plasticity-like mechanisms and so may contaminate the effects of an rTMS protocol. In the present study, we explored the effect of anesthetic on MEP amplitude, recorded before and after intermittent theta burst stimulation (iTBS), a patterned rTMS protocol with reported facilitatory effects. MEPs were assessed in the brachioradialis muscle of the upper forelimb under two anesthetics: a xylazine/zoletil combination and urethane. We found MEPs could be induced under both anesthetics, with no differences in the resting motor threshold or the average baseline amplitudes. However, MEPs were highly variable between animals under both anesthetics, with the xylazine/zoletil combination showing higher variability and most prominently a rise in amplitude across the baseline recording period. Interestingly, application of iTBS did not facilitate MEP amplitude under either anesthetic condition. Although it is important to underpin human application of TMS with mechanistic examination of effects in animals, caution must be taken when selecting an anesthetic and in interpreting results during prolonged TMS recording.

## Introduction

Transcranial magnetic stimulation is a technique which exploits Faraday’s law of electromagnetic induction to induce a current in the brain using a magnetic field generated by a coil. This technique is applied either as a single pulse, in order to transiently depolarize an underlying area of neural tissue, or repetitively, where pulses are delivered with a defined frequency and pattern. There has been increasing interest in the application of repetitive transcranial magnetic stimulation (rTMS) because of its potential to alter corticospinal excitability, namely the probability that cells in a targeted area will fire in response to an excitatory input. Such changes can be brought about through synaptic plasticity, the ability of synaptic connections to either strengthen or weaken in response to external or internal stimuli (Ho et al., 2011). A major benefit of rTMS compared to traditional, electrical brain stimulation is that it is non-invasive (Vallence and Ridding, 2014), however, the mechanisms by which rTMS exerts its effect on the brain are still poorly understood (Muller-Dahlhaus and Vlachos, 2013; Tang et al., 2015).

Clinically, rTMS has been applied as an experimental treatment for a wide variety of neuropsychiatric and neurophysiological disorders including stroke, depression, and tinnitus, with variable effectiveness (Dell’osso et al., 2011; Lefaucheur et al., 2014). The measured success of rTMS may be due to the variety of potential rTMS stimulation parameters. There are an unquantifiable number of possible combinations of location, intensity, pattern, and duration of the stimulus train as well as many possible configurations of the electromagnetic coil, and we currently lack sufficient experimental data to understand how these stimulation parameters map to outcomes in different conditions. In the relatively short history of rTMS, it has broadly been understood that low frequency rTMS (around 1 Hz) is inhibitory (Chen et al., 1997) and higher frequencies appear to be facilitatory (Fitzgerald et al., 2006; Ziemann et al., 2008), although significant variation is reported. Attempts to improve the strength and reproducibility of effects has led to recent application of patterned stimulation protocols such as theta burst stimulation (TBS) and quadripulse stimulation (QPS). These protocols have shown some capacity to modulate corticospinal excitability (Huang et al., 2005; Hamada et al., 2008), although reproducing the effects originally reported with TBS has been difficult (Brownjohn et al., 2014). Both TBS and QPS protocols include variants that can putatively drive either facilitation [such as intermittent TBS, intermittent theta burst stimulation (iTBS); or 5 ms interstimulus interval QPS] or inhibition (such as continuous TBS, cTBS; or 50 ms interstimulus interval QPS).

In an effort to narrow the parameter space and maximize the ability of rTMS protocols to induce plasticity of neural responses, a variety of investigations have been made, primarily by indirect means in humans but also more directly in animal models. In humans, the efficacy of rTMS protocols is usually measured by non-invasively stimulating the motor cortex with single pulses of TMS, before and after an rTMS protocol, and recording the motor evoked potential (MEP) from target muscles using electromyography (EMG; Jennum et al., 1995; Di Lazzaro et al., 2004). Changes in the amplitude of the MEP after rTMS provide a measure of the change in excitability of the polysynaptic neural pathway descending from the motor cortex to the target muscle(s) (Fitzgerald et al., 2002; Huang et al., 2005; Hamada et al., 2007). In contrast, studies of rTMS effects in animal models have focused on cellular and molecular markers of neuroplastic change rather than modulation of MEPs (Gersner et al., 2011; Muller-Dahlhaus and Vlachos, 2013; Volz et al., 2013). This approach offers researchers the chance to observe the direct effects of rTMS on stimulated neurons and to understand the mechanisms through which rTMS influences those neurons. However, despite significant progress, how molecular changes measured very close to the site of stimulation in animals relate to changes observed in complex, polysynaptic MEPs measured in humans is as yet unknown.

In order to bridge the gap in knowledge between single cell and molecular changes measured in animals and network level changes seen in humans, an interim step of correlating molecular changes with MEPs recorded in animals may be of value. This would enable meaningful comparison of protocols in animals and humans that would support feedback between preclinical testing and human research. However, unlike human studies, MEPs in animal studies are often elicited under anesthesia (Luft et al., 2001; Rotenberg et al., 2010; Hsieh et al., 2015) because of practical difficulties with eliciting MEPs in awake animals without significant restraint (Linden et al., 1999). It is well known that different anesthesia compounds have differing effects on cortical excitability, and so may impact on plasticity induction by an rTMS protocol. However, with the exception of a single study comparing pentobarbitone and a ketamine/xylazine combination in rats (Vahabzadeh-Hagh et al., 2011), information about the effects of different anesthetics on MEP amplitude and resting motor threshold (RMT) is currently lacking. Before this molecular-network gap can be bridged, then, it is necessary to evaluate the effect these anesthetic compounds have on MEPs.

In the present investigation we compared two injectable rodent anesthetics, xylazine/zoletil, recently used by Hsieh et al. (2015) to study the modulation of MEPs in rodents using iTBS, and urethane, at doses our lab commonly uses to measure synaptic plasticity in the brain (Reynolds et al., 2001, 2004; Schulz et al., 2010). We compared how these agents influence basic components of MEPs in rats and how they responded to rTMS.

## Materials and Methods

### Animal Preparation

Experiments were conducted on 27 Male Wistar rats (250–350 g), sourced from the Hercus Taieri Resource Unit of the University of Otago. All experiments and procedures were approved by the Animal Ethics Committee of the University of Otago (protocol number 77/13). Animals were group housed in standard cages under a 12 h/12 h light-dark cycle. The room was maintained at 22°C with constant humidity. Water and food was available *ad libitum* between experimental procedures.

On the day of recording, rats were deeply anesthetized with either urethane (Sigma–Aldrich, New Zealand; 1600 mg/kg, I.P.; *n* = 13) or a combination of xylazine (Bayer, New Zealand; 10 mg/kg, I.P) and zoletil (Zoletil 100, Virbac New Zealand; 80 mg/kg, I.P; *n* = 14). Anesthesia depth was monitored periodically using the pedal withdrawal (“toe-pinch”) reflex at the same relative timing and frequency in all animals. Absence of the reflex indicated that a standardized depth of anesthesia and analgesia was present and was maintained during electrode implantation and recording. Increments of approximately 1/3 of the original dose of urethane or zoletil were given prior to the commencement of recording to maintain the absence of the pedal withdrawal reflex, i.e., the absence of any visible movement in muscles in the lower leg, upper leg or abdomen following the pedal stimulus (seen in approximately 50% of animals at that stage). Pedal withdrawal was monitored in this manner and remained absent prior to the commencement of stimulation and after the baseline of MEPs was recorded. The interval between application of additional anesthetic doses and TMS measurements was comparable between the drugs: 51 ± 24 min for urethane (mean ± SD) and 38 ± 9 min for zoletil (mean ± SD). There was no significant correlation between the number of additional anesthetic doses given and RMT (*R*^2^ = 0.1533, *p* > 0.05) or baseline MEP amplitude (*R*^2^ = 1.1 × 10^-6^, *p* > 0.05). The pedal stimulus was administered at least 1 min away from any TMS stimulation to avoid inadvertent paired associative stimulation effects.

Temperature was maintained at 37°C using a heating pad and rectal thermometer. Rats were placed in a grounded stereotaxic frame (Narishige, Japan) and electrically isolated from metal ear bars using parafilm.

### Electromyographic (EMG) Recording

To record MEPs, silver-wire electrodes were fashioned from 26 gage needles soldered to steel wire. These were placed in the right brachioradialis muscle, located via palpation. Reference electrodes were placed between the third and fourth digits in the paw of the same forelimb. A ground electrode was placed subcutaneously above the tail. The right forepaw was immobilized using surgical tape adhered to the mat on which the rats were placed. The signal was amplified 1000 times using an Axoclamp900a (Molecular Devices, Sunnyvale, CA, USA), digitized using a Digidata 1322a (Molecular Devices, Sunnyvale CA, USA) and recorded using pClamp 10 software (Molecular Devices, Sunnyvale CA, USA). The signal was sampled at 10 kHz with no bandpass filtering. Successive MEPS were not excluded on the basis of amplitude variability, however, episodes showing a latency shorter than 4 ms were rejected (see Data Analysis). To ensure stability of recording, interaction with the animal or experimental set up was prohibited during active stimulation and recording.

### Transcranial Magnetic Stimulation

A 25 mm air-cooled figure-of-eight coil (Magstim) was placed over the rat’s scalp and positioned to maximally activate the underlying M1 area unilaterally. The center of the coil was placed over the left motor cortex then rotated marginally in the antero-posterior direction anti-clockwise in the same manner as Rotenberg et al. (2010), in order to aid in producing MEPs with maximum amplitude. The coil was powered by two Magstim Rapid^2^ stimulators. Pulses were delivered at 0.2 Hz beginning at ∼65% machine output to determine optimum stimulation location, assessed by observing evoked responses. When coil location was optimized, fine adjustment of electrode placement was made, until a distinct MEP was observed. RMT was determined by decreasing stimulator output by 5% until MEPs vanished and then increasing in 1% increments until 6 MEPs of ≥50 μV peak to peak, were elicited out of every 12 TMS pulses. 20 min of baseline MEPS were recorded every 5 s (0.2 Hz) at 120% of RMT.

Intermittent theta burst stimulation (X/Z *n* = 6; urethane *n* = 7), or sham stimulation (X/Z *n* = 8; urethane *n* = 6) was then applied for a total duration of 142 s. iTBS consists of a burst of three pulses at 50 Hz, repeated at 5 Hz, delivered for 2 s with an 8 s off period (Huang et al., 2005). Four hundred and fifty pulses were delivered based on previous data from our lab using electrical iTBS which showed a change in synaptic efficacy elicited using this protocol (Barry et al., 2014). rTMS was delivered at 80% of RMT (up to 50% of machine output, the maximum intensity the Rapid^2^ can deliver iTBS) for 450 pulses and a final 20 min of MEPs post stimulation recorded at 0.2 Hz at 120% RMT immediately after verum or sham treatment. Sham stimulation was delivered by unplugging the verum coil at the stimulator and plugging in another coil held at least 2 m from the rat head whilst the iTBS protocol was run, in order to mimic auditory stimulation and maintain verum coil positioning.

### Data Analysis

All MEP data were analyzed oﬄine using Axograph X version 1.5^1^ Each minute run was composed of 12 individual sweeps, all of which were successful at evoking MEPs at 120% of motor threshold, and amplitudes were measured from peak-to-peak. For each animal, all data were normalized to the final 5 min of baseline amplitude recordings and expressed as a percentage change, to allow for between-subject comparison. Normalized amplitudes were then grouped into 2-min bins and a final 3-min bin. Latencies were measured for each sweep from the onset of the TMS artifact, and waveforms with latencies < 4 ms were eliminated (∼30% of animals excluded). These were excluded to ensure that any MEPs induced by direct activation of the spinal circuitry by the coil were not included in the analysis. Remaining traces in each minute run were averaged and the peak-to-peak amplitude measured. RMT and baseline amplitudes were compared between the two groups using students unpaired *t*-tests. Data were determined to be normally distributed by the D’Agostino-Pearson omnibus normality test. Anesthetic and stimulation effects on normalized amplitudes were compared using two-way repeated measures ANOVA. All data were analyzed using Graphpad Prism 7 and expressed as mean ± standard deviation, unless otherwise indicated.

## Results

### Resting Motor Threshold and Baseline Amplitude Measured in the Brachioradialis Were Not Affected by Anesthetic

**FIGURE 1:**
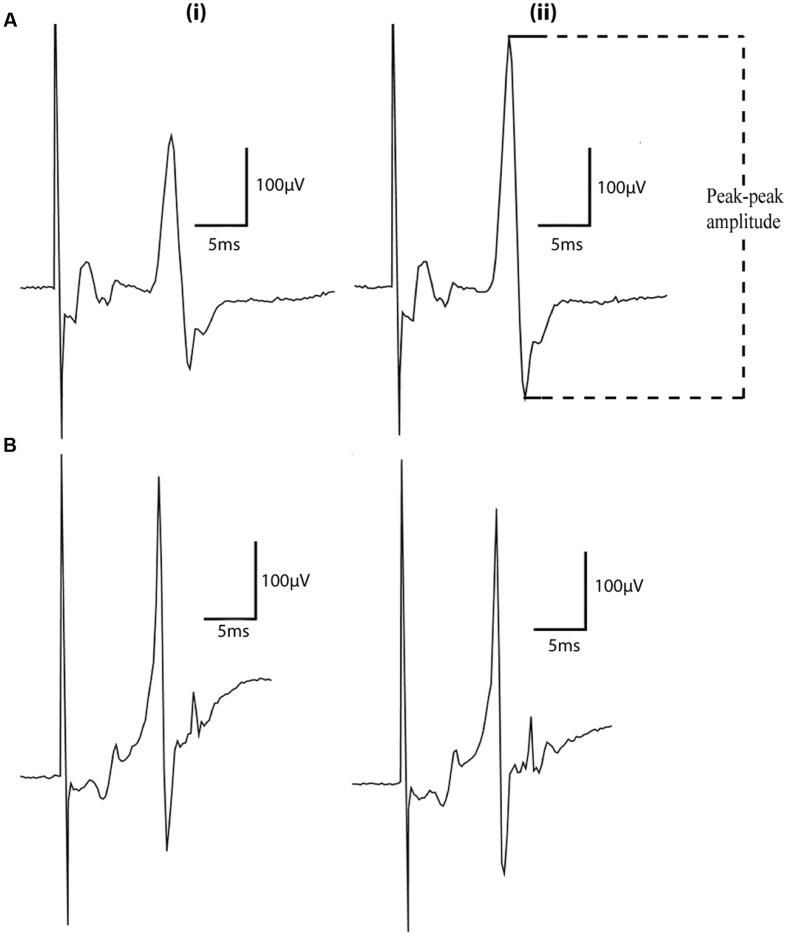
**Representative motor evoked potentials (MEP) traces recorded in animals anesthetized with (A) xylazine/zoletil or (B) urethane before (i) and after (ii) intermittent theta burst stimulation (iTBS).** Traces shown are an average of 12 sweeps. The dashed lines indicate points used for peak-to-peak measurement of MEP amplitudes.

**FIGURE 2:**
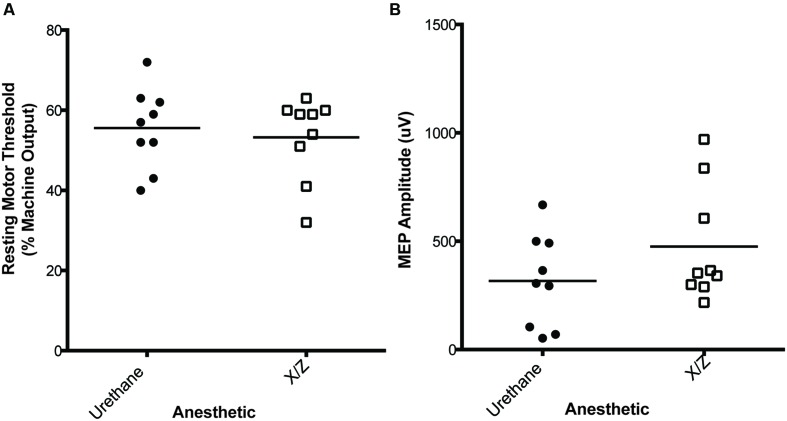
**No differences were observed in resting motor threshold (RMT) (A) or baseline amplitude (B) in the MEPs recorded from the brachioradialis muscle in rats anesthetized with urethane (*n* = 9) or xylazine/zoletil (*n* = 9).** Baseline amplitude was averaged from MEPs recorded in final 5 min of pre-iTBS baseline. X/Z = xylazine/zoletil.

Representative MEP traces evoked in animals under both anesthetics can be seen in **Figure 1** RMT (% of machine output) was measured in the right brachioradialis muscle at 55.6% ± 10.0% for animals anesthetized with urethane (*n* = 9) and for rats anesthetized with xylazine/zoletil 53.2% ± 10.4% (*n* = 9; **Figure 2A**) (mean ± SD). Under the same conditions, the baseline amplitudes from the final 5 min of the baseline recording period were compared at a stimulus intensity of 120% machine output and measured as 316 ± 213 μV for urethane and 475 ± 266 μV for xylazine/zoletil (**Figure 2B**). There were no significant effects of anesthetic on RMT (*t*_16_ = 0.48; *p* > 0.05) or baseline amplitude (*t*_16_ = 1.39; *p* > 0.05).

### MEP Amplitudes Increase across Baseline Recording Period in Animals Anesthetized with Xylazine/Zoletil But Not Urethane

**FIGURE 3:**
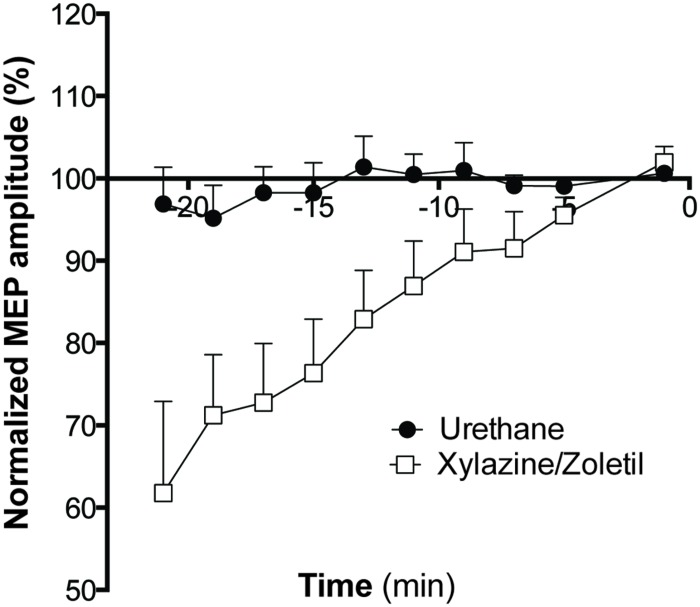
**The effect of anesthetic on MEP amplitude during the baseline period.** MEPs recorded from all animals anesthetized with xylazine/zoletil (*n* = 9), showed increased amplitudes over the baseline recording period, however, those from urethane anesthetized animals (*n* = 9) remained stable. Data shown are mean ± SEM. Normalized amplitudes from each minute are grouped into 2-min bins.

Responses recorded from animals anesthetized with xylazine/zoletil increased in amplitude over the full baseline recording period (**Figure 3**). This contrasted markedly to the baseline responses evoked in urethane-anesthetized animals. Two way ANOVA with repeated measures showed a significant interaction (Greenhouse-Geisser corrected, *F*_2,38_ = 5.445; *p* < 0.01), indicating that the amplitudes in the two groups changed differently over time. A significant main effect of TIME (Greenhouse-Geisser corrected, *F*_2.3,38_ = 8.1; *p* < 0.01) was also revealed.

The effect of time between anesthetic dose and commencement of recording (average of 43 min) on the stability of MEP baselines was examined in the xylazine/zoletil group. Linear regression showed no significant relationship between this wait time and the slope of the normalized amplitudes of the baseline recording in the same animal (*F*_1,7_ = 0.456, *R*^2^ = 0.061, *p* > 0.05).

### Intermittent Theta Burst Stimulation Did Not Significantly Alter MEP Amplitudes in Rats Anesthetized with Xylazine/Zoletil or Urethane

**FIGURE 4:**
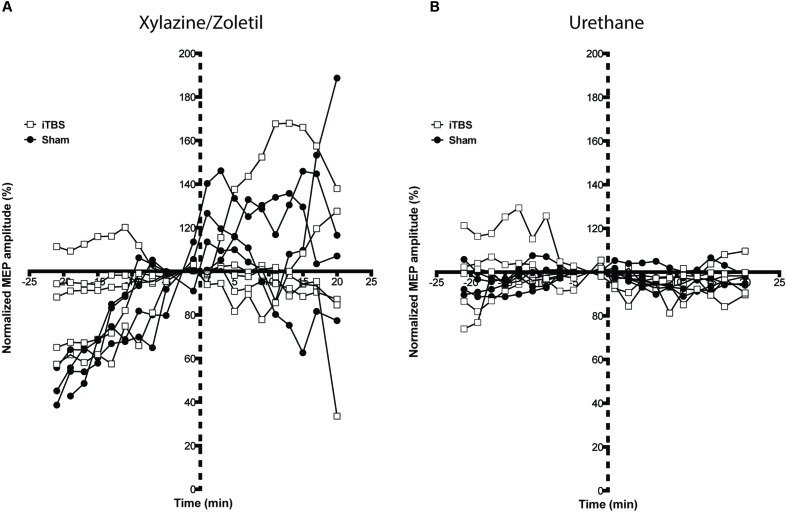
**Motor evoked potentials recorded from individual animals under each anesthetic, with both iTBS and sham animals shown together.** Variability between animals anesthetized with **(A)** xylazine/zoletil is greater than in animals anesthetized with **(B)** urethane. A rising amplitude over the baseline recording period can be seen in most animals in **(A)**. Two rats show a moderate rising amplitude whereas a single rat shows a falling baseline. Stimulated animals are shown with squares; sham animals with filled circles. Dashed axis line indicates Sham or iTBS treatment (time point 0).

**FIGURE 5:**
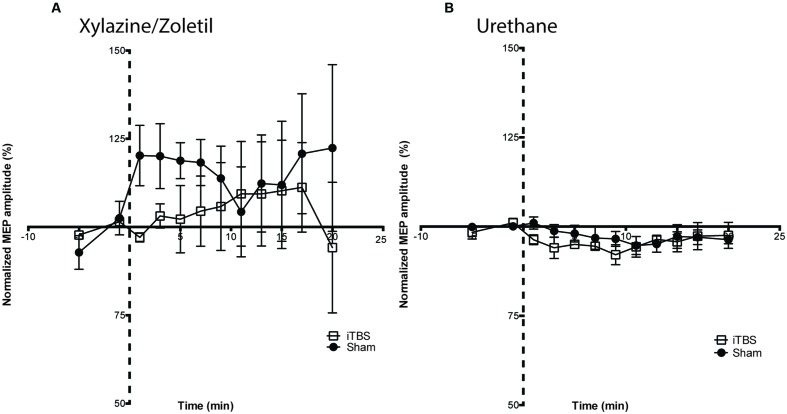
**Intermittent theta-burst stimulation did not significantly affect MEP amplitudes recorded in animals anesthetized with either (A) xylazine/zoletil (iTBS = 5; Sham = 4) or (B) urethane (iTBS = 5; Sham = 4).** Note the baseline data are truncated to 5 min preceding the protocols. All data shown are mean ± SEM. Normalized amplitudes from each minute are grouped into 2-min bins. Dashed axis line indicates Sham or iTBS treatment (time point 0).

**Figure 4** shows the recordings made under each type of anesthetic identified by whether iTBS (squares) or sham (circles) was the protocol. Responses under xylazine/zoletil increased over the baseline in all but one animal, and continued in many animals following the protocol, regardless of whether administered iTBS or sham stimulation (**Figure 4A**). The increasing baseline pattern seemed to level off in a number of animals during the 10 min following the protocol, giving the impression in a group-averaged graph (**Figure 5A**) that MEPs were potentiated following the baseline period, especially so when the baseline period was truncated to 5 min preceding the protocol. In contrast, **Figure 4B** shows the lack of systematic MEP baseline change under urethane and the stable responses throughout both iTBS and sham under this anesthetic (see group averaged responses in **Figure 5B**). The fact that this was not due to the effects of TMS is apparent when considering that the sham animals showed a greater ‘pseudo potentiation’ effect under xylazine/zoletil than those administered iTBS. Although the appearance is that iTBS treatment transiently suppressed the rising baseline, data at these points were not significantly different.

Statistical comparisons of the effects of the protocols on animals under both anesthetics were performed using a two-way ANOVA with repeated measures, with STIMULATION and TIME as the factors. Under xylazine/zoletil anesthesia, there was no significant difference as a result of TIME (Greenhouse-Geisser corrected, *F*_2.5,17_ = 0.69; *p* > 0.05) or TIME^∗^STIMULATION interaction (Greenhouse-Geisser corrected, *F*_2.5,17_ = 0.69; *p* > 0.05) (**Figure 5A**). Under urethane anesthesia there were no significant differences as a result of TIME (Greenhouse-Geisser corrected, *F*_3.2,23_ = 2.17; *p* > 0.05) or TIME^∗^STIMULATION interaction (Greenhouse-Geisser corrected, *F*_3.2,23_ = 0.84; *p* > 0.05).

## Discussion

With the widespread use of MEPs as a measure of efficacy in human TMS studies, the present investigation aimed to explore the effect of anesthetics on MEPs, as a potential complicating variable in mechanistic studies performed on animals. We compared two anesthetics, one that has been previously used to study TMS modulation of MEPs in rodents, xylazine/zoletil (Hsieh et al., 2015), and another that has frequently been used to study the excitability of single neurons and synaptic plasticity at monosynaptic connections, urethane (Reynolds and Wickens, 2000; Clement et al., 2008; Parr-Brownlie et al., 2009; Schulz et al., 2009; Wilson et al., 2010; Harris et al., 2014). To our knowledge urethane has not been used to study TMS effects on MEPs. We found that in animals anesthetized with urethane, it was possible to elicit MEPs over an extended baseline period, which remained stable. In contrast xylazine/zoletil, under the same recording conditions and baseline duration, showed a marked systematic baseline increase in responses. With both agents, levels of anesthesia were adjusted in each animal to the same suppression of peripheral responsiveness, i.e., the absence of a pedal reflex. With both agents, there was no induced modulation of MEP amplitudes when comparing the effects of an iTBS rTMS protocol with a sham protocol. However, there were marked qualitative differences in how MEPs ‘responded’ following the baseline period under both anesthetics.

### Use of Anesthetics to Study Effects of TMS in Animals

Characterization of the effect of neuroactive compounds on TMS-evoked measures has been extensively discussed previously (Ziemann et al., 2015). In humans, motor threshold is elevated by sodium channel blockers (Ziemann et al., 1996; Boroojerdi et al., 2001) as well as by certain NMDA-receptor antagonists (Wohlfarth et al., 2000). Even markers of plasticity, such as BDNF, are different after rTMS treatment in anesthetized vs. awake animals (Gersner et al., 2011). Hence the decision to use an anesthetic and the agent of choice must be carefully made, for the results of TMS studies in animals to be generalized to humans.

In studies using MEPs as a measure of cortical excitability in rodents, anesthetics are frequently used, to avoid the neural and systemic stress effects of restraint. In the few studies investigating magnetically induced MEPs in animal models, there has been a great variation in anesthetic choice. These range from inhalants such as isoflurane and halothane (Luft et al., 2002; Deffeyes et al., 2015; Vinit et al., 2016), and injectable anesthetics such as sodium pentobarbital (Rotenberg et al., 2010; Vahabzadeh-Hagh et al., 2011), and xylazine/zoletil (Hsieh et al., 2015). All anesthetics are known to impact on neuronal function to varying degrees, with their exact mechanisms of action altering how this impact manifests (Angel and Gratton, 1982; Anis et al., 1983; Simons et al., 1992; Lahti et al., 1999; Antkowiak, 2002; Antunes et al., 2003). Xylazine, for example, is an α2-adrenoreceptor agonist (Greene and Thurmon, 1988), primarily used for muscle relaxation and sedation (Ko, 2013). It is also used in combination with other compounds such as ketamine or zoletil (Ferrari et al., 2005; Ko, 2013), because of its potentiating effects on anesthetic and analgesic effects. When used in conjunction with ketamine anesthesia, the effect is reportedly variable between animals (Green et al., 1981; Smith, 1993). Zoletil is itself a mix of tiletamine and zolazepam (Ko, 2013). Tiletamine is an NMDA-receptor antagonist, chemically related to ketamine and acts by fundamentally the same mechanisms, although it is more potent. Zolazepam is a benzodiazepine, a positive modulator of the GABA-A receptor, acting to decrease neuronal excitability (Griffin et al., 2013). Action at the NMDA receptor might potentially influence rTMS-induced cortical plasticity, which has been shown to require NMDA activation (Huang et al., 2007; Labedi et al., 2014). However, the demonstration of rTMS-induced plasticity by Hsieh et al. (2015), namely potentiation of MEPs after iTBS in healthy rats, has provided some evidence that iTBS-induced facilitation is observable using xylazine/zoletil.

In contrast to the relatively targeted effects of xylazine/zoletil, urethane is an anesthetic with non-specific actions potentially affecting multiple neurotransmitter systems (Hara and Harris, 2002). Its use is restricted in many countries to non-survival experiments, due to its prominent carcinogenic effects and long duration of action. The most prominent effect on the nervous system is a reduction of excitatory transmission with a minimal enhancement or no effect on GABA transmission (Evans and Smith, 1982; Dalo and Hackman, 2013). The main advantage of the use of urethane in neural recording studies, is the duration of anesthesia produced and the stability of anesthesia it induces. Using electrical stimulation, we have previously observed clear TBS-induced potentiation and depression of synaptic circuits using urethane at the doses used here (Barry et al., 2014), suggesting that urethane does not abolish the type of brain plasticity likely to be observed following TBS applied with rTMS (Maggi and Meli, 1986).

For the combination of zoletil with xylazine, previous literature has suggested a range of 40–65 mg/kg for zoletil in rats (Silverman et al., 1983; Ferrari et al., 2005). Anesthetic duration has been suggested to last for anywhere from 1 to 4 h in combination (Wilson et al., 1992; Ferrari et al., 2005). Using 65 mg/kg of zoletil and 10 mg/kg of xylazine for induction, Hsieh et al. (2015) reported deep anesthesia for ∼4 h. Interestingly, our initial attempts at using 65 mg/kg zoletil with 10 mg/kg xylazine failed to bring rats to an adequate plane of anesthesia, resulting in our application of a 20% greater dose of 80 mg/kg for zoletil. This raises the possibility that differences in the level of animal sedation between studies may have an impact on the size of MEPs and also may critically influence the ability to induce MEP plasticity using rTMS.

### The Rise in MEP Amplitude during Baseline Recording Period in Xylazine/Zoletil Anesthetized Rats

During the baseline recording period, MEP amplitudes consistently increased across time in rats anesthetized with xylazine/zoletil. This effect was seen in the majority of animals anesthetized with this agent combination, and contrasted with urethane in the hands of the same experimenter, where MEP amplitude remained relatively stable across the baseline. This strongly suggests that the anesthetic agent was the underlying cause. One possible mechanism of the increasing MEP baseline amplitudes may be the actions and metabolism of the NMDA-receptor antagonist component of zoletil, tiletamine. Metabolism of tiletamine varies between species, with indications of a half-life of 1 h in dogs and 2 h in cats (Ko, 2013), though little information exists on the process in laboratory rodents. In its zoletil form (given at up to 60 mg/kg), it induces a maximum sleep time of just under 2 h in rats (Wilson et al., 1992). At the concentrations used here, it is likely that the tiletamine action initially elevate the RMT, defined earlier as the stimulator output necessary to elicit MEPs >50 μVs, but potentially this elevating effect may have reduced over time as tiletamine was metabolized. With progressive attenuation of the NMDA-receptor antagonism, physiological RMT would have progressively decreased, manifesting as a slow increase in MEP size. We also cannot exclude a contribution of the muscle relaxant effect of xylazine wearing off. Xylazine was given as a single dose, however, its half-life is up to 2–3 h (Garcia-Villar et al., 1981; Veilleux-Lemieux et al., 2013). Since recordings were completed by approx. 100 min after xylazine induction in all animals, an effect on MEPs, if any, of metabolism of xylazine would therefore more likely have occurred in the second half of recording rather than during the baseline.

The sloping baseline MEP amplitude may also have been influenced by the time between anesthetic injection and commencement of the recording procedures. Hsieh et al. (2015) did not report a sloping baseline and stated they waited 60 min from injection to starting the recording experiment, whereas we waited an average of 43 min following induction. Despite the small difference between waiting 60 and 43 min, the additional metabolism of tiletamine in that time may constitute a possible reason for the disparities in the results of Hsieh and our own. Linear regression showed no association between the baseline slope of MEP amplitudes and the wait time for each animal. These increases in MEP amplitudes also appear in all of the TBS-exposed MEP recordings in Hsieh et al. (2015), however, the effect is less dramatic, with only two baseline points shown. These data, taken together, demonstrate the importance of applying protocols that might modulate MEPs in anesthetized animals at a point when all baseline anesthetic effects have stabilized, and to ensure that all extended baseline data (beyond 10 min preceding the protocol) are shown in these graphs.

### Intermittent Theta Burst Stimulation Did Not Affect MEP Amplitudes in Rats Anesthetized with Xylazine/Zoletil or Urethane

The present study failed to find any changes in MEP amplitude after iTBS, in contrast to that reported by Hsieh et al. (2015) under xylazine/zoletil anesthesia. In rats, iTBS has previously been shown to induce changes in markers of inhibitory and excitatory neuronal activity, as well as increases in the learning rate in an associative tactile task (Mix et al., 2010; Hoppenrath and Funke, 2013; Labedi et al., 2014). Changes in MEP amplitude after iTBS have been demonstrated in humans (Huang et al., 2005; Ziemann et al., 2008; Nettekoven et al., 2014), however, to our knowledge have only been shown once in rats (Hsieh et al., 2015). There are a few methodological differences between the current study and that reported by Hsieh et al. (2015) that may have contributed to the result, at least for our experiments using xylazine and zoletil. Firstly, in our study we applied TBS protocols using 450 pulses compared to the 600 pulses used by Hsieh et al. (2015). This was based on our previous work using urethane, where we showed clear differential synaptic plasticity using 450 pulses applied using electrical stimulation (Barry et al., 2014). Previous work in humans has shown that variations in pulse number using the same rTMS protocol may significantly affect the plasticity-inducing effects of those patterns (Gamboa et al., 2010; Nettekoven et al., 2014). However, the effect of increasing pulse number is usually manifest by an increase in the duration of the after effects on MEPs rather than an alteration in the degree of change, with even 300 pulses inducing a significant effect on MEPs lasting for 20 min (Huang et al., 2005). In addition, as described in the previous section, there may have been a suppressive effect on baseline MEPs related to the zoletil. However, even in individual experiments that showed minimal sloping baseline effect, iTBS had no potentiating effect that could be distinguished from sham animals. Though the amount of xylazine/zoletil given in this study was quantitatively higher, possibly indicating these rats were under a deeper level of anesthesia, the same measures of anesthetic depth were used to inform dose (i.e., pedal withdraw reflex). The fact that MEPs in urethane-anesthetized rats showed a stable baseline but were not affected by iTBS together raises the possibility that iTBS applied using rTMS for 450 pulses does not produce any plastic responses of MEPs, although this number of pulses applied electrically can induce plasticity at synaptic connections in the brain (Barry et al., 2014).

While we expected to demonstrate an effect of iTBS on MEPs based on the findings of several human and animal experiments with small numbers of participants, the lack of iTBS effects in our study is in line with at least one study using larger numbers of human participants. The first study to describe iTBS (and cTBS) -evoked changes in MEPs (Huang et al., 2005) involved nine participants in a repeated measures design. A larger study by Hamada et al. (2013) however, found that with many more participants (*n* = 56), response to iTBS measured with MEPs was extremely variable between subjects and the mean response to iTBS across participants was essentially zero. A similar result was reported by Lopez-Alonso using 56 subjects (Lopez-Alonso et al., 2014). A recent paper (Héroux et al., 2015) reported findings from a survey of 47 researchers who work with TMS. They state that only 45% of those surveyed could reproduce the original results using iTBS. Why there exists such a large inter-individual variability in iTBS neuromodulation is still unknown, but may be rooted in the sheer number of factors which influence TMS-induced plasticity, such as genetics, age and circadian cycle (Ridding and Ziemann, 2010; Hamada et al., 2013). Indeed, Héroux noted that 70% of survey-responders reported knowing colleagues who screened subjects according to some of the factors known to affect rTMS-induced plasticity to increase chances of responding favorably to stimulation protocols.

## Conclusion

We have demonstrated in this study that a xylazine/zoletil anesthetic combination induces a rising baseline in MEP amplitude, potentially related to the metabolism of the compounds. In designing any animal study, care should be taken with anesthetic choice and adequate levels of sedation in order to limit the often multi-factorial effects these agents have on plasticity. Additionally, this study highlights and reconfirms the variability of stimulation protocols, in this case iTBS, something reported more often now in the human literature.

## Author Contributions

Experimental background and design: JNR, JS, PB, and NM. Data collection and analysis: MS, NM, and JNR. Troubleshooting and methodological advice: AT and JR Wrote the manuscript: MS and JNR. Revised manuscript and critical review: All authors.

## Conflict of Interest Statement

The authors declare that the research was conducted in the absence of any commercial or financial relationships that could be construed as a potential conflict of interest.

## Footnotes

1 www.axograph.com
